# The multifaceted role of E3 ubiquitin ligases in cancer metastasis: mechanisms, targets, and therapeutic implications

**DOI:** 10.1042/EBC20253037

**Published:** 2025-12-12

**Authors:** Meghna Singh, Akshita Upreti, Samit Chattopadhyay, Manas Santra

**Affiliations:** 1 Department of Biological Sciences, BITS – Pilani, K.K. Birla Goa Campus, Sancoale, Goa, 403726, India; 2 National Centre for Cell SciencePune, Maharashtra, 411007, India

**Keywords:** E3 ubiquitin ligases, metastasis, PROTACs, therapeutics

## Abstract

Cancer metastasis is one of the hallmarks of cancer. This multistep process involves a cascade of alterations at the cellular and molecular level, including the epithelial-to-mesenchymal transition (EMT), invasion, migration, extracellular matrix (ECM) degradation, angiogenesis, and colonization. Expression level of critical factors associated with these processes is altered at the post-translational level through ubiquitination. Therefore, E3 ubiquitin ligases, components of the ubiquitin-mediated proteasome system, play a crucial role in controlling each step of metastasis by promoting the ubiquitination of several important factors. In this review, we have summarized the importance of E3 ligase in metastasis. Several E3 ligases act as promoters, while others act as repressors of metastasis. This article focuses on the potential role of E3 ubiquitin ligases in cancer metastasis and reveals their molecular function and targets, which are crucial for therapeutic interventions in anti-cancer therapies. Further, we covered the development of small molecule inhibitors and proteolysis-targeting chimeras to target E3 ubiquitin ligases involved in promoting metastasis for therapeutic intervention. Despite tremendous advancements, there are still many unanswered questions, especially regarding the complete characterization of the diverse range of E3 ligase functions and the conversion of preclinical discoveries into successful clinical treatments. In addition, future directions are concentrated on using technologies to develop highly specific therapeutic interventions and exploring their potential in combination with other treatment modalities, including immunotherapy, to ultimately overcome the challenges of cancer metastasis.

## Introduction

E3 ubiquitin ligases are the key components of the ubiquitin–proteasome system (UPS) and are primarily known for their role in the ubiquitination of proteins to regulate protein homeostasis. The UPS system includes ubiquitin-activating enzymes (E1), ubiquitin-conjugating enzymes (E2), ubiquitin ligases (E3), and proteasome. Ubiquitin is a small 76-amino acid protein, covalently attached to target proteins through an isopeptide bond, and serves as a signal for degradation by the 26S proteasome or protection from degradation.

The E3 provides facilitation of the transfer of activated ubiquitin on the target proteins. The E3 ligases associated with various cellular processes, including cell proliferation, cellular signaling, cell deaths, genome stability, epithelial-to-mesenchymal transition (EMT), migration, invasion, and angiogenesis through controlling protein homeostasis. Therefore, deregulation of E3 ubiquitin ligases is closely associated with several diseases including cancer, and interaction with the tumor microenvironment. The diverse roles of E3 ligases and their involvement in multiple stages of metastasis indicate a complex regulatory network that must be dissected to identify key players. This review aims to provide an up-to-date analysis of the roles of E3 ubiquitin ligases in cancer metastasis, focusing on their mechanisms, targets, and therapeutic potential, while also identifying research.

### E3 ubiquitin ligases

E3 ubiquitin ligases comprise a large family of around 600 proteins in humans [1]. Based on their catalytic mechanisms and respective structural domains, they are broadly classified into four categories: RING-finger type (really interesting new gene), HECT type (homologous to the E6-AP carboxyl terminus) domain ligases, U-box type, and RBR (RING-in-between-RING) type. All E3 ubiquitin ligases function by associating with an E2 ubiquitin-conjugating enzyme and specific substrate proteins that are targeted for ubiquitination. However, they differ in how they transfer ubiquitin from the E2 enzyme to the substrate. RING-finger and U-box E3 ligases facilitate a direct, single-step transfer of ubiquitin from the E2 enzyme to the substrate [2]. In contrast, HECT-type and RBR E3 ligases use a two-step mechanism: first, ubiquitin is transferred from the E2 enzyme to the E3 ligase itself, and then from the E3 to the substrates. Despite differences in structure and mechanism, dysregulation of E3 ligases is commonly linked to various diseases, including cancer, underscoring their critical role in maintaining cellular homeostasis.

Among these E3 ubiquitin ligases, RING domain E3 ligases, the most abundant class, primarily function as scaffolds that bring the E2 ubiquitin-conjugating enzyme and the substrate protein into close proximity to facilitate the direct transfer of ubiquitin from the E2 to the substrate protein. RING-finger type could be monomeric, including the c-CBL, CBL-b, and CBL-c family, and multimeric complexes like anaphase-promoting complex (APC/C) and Cullin ring ligase (CRL) complexes.

CRL complexes are composed of a scaffold, Cullin protein, a RING protein (Rbx1 or Rbx2), and a substrate receptor (SR) unit, often with an adaptor protein. The human genome encodes eight different Cullin (Cul) proteins (Cul1, Cul2, Cul3, Cul4A, Cul4B, Cul5, Cul7, and Cul9), which serve as the core organizers of these complexes and determine their distinct subclasses. The first identified CRL1, the SCF E3 ligase, uses SKP1 as an adaptor to connect the Cul1 scaffold to an F-box substrate receptor. Among the CLRCs, SCF (SKP1, Cullin1, F-box protein) complexes are well studied. The human genome codes for 69 F-box proteins that confer the complex with its diverse substrate recognition and specificity. SCF ligases catalyze the ubiquitination of substrates and regulate various cellular processes, including the cell cycle and DNA damage repair [3].

For CRLs based on Cul2 or Cul5, the adaptor proteins are Elongins B and C, which mediate the linkage between the N-terminal of the Cullin and the BC-box substrate receptors. A prominent substrate of the CRL2 complex is hypoxia-inducible factor 1α (HIF-1α), which plays a key role in the hypoxia response. CRL5 has similar components but uses RBX2 as its RING subunit and is primarily involved in signaling transduction, viral infections, and tumorigenesis [4,5]. The CRL3 complex is distinguished by its substrate receptors, typically BTB/POZ domain proteins, which directly interact with the Cul3 scaffold protein, eliminating the need for an adaptor protein. The BTB domain, often associated with MATH and Kelch domains, is responsible for substrate recognition. CRL3 plays a crucial role in regulating cellular homeostasis, the oxidative stress response, and tumorigenesis [6].

CRL4 complexes include two homologous Cullin proteins, Cul4A and Cul4B, that share a common adaptor protein, damage-specific DNA-binding protein 1 (DDB1), and DDB1/CUL4-associated factors (DCAF) as substrate receptors [7]. These complexes have a significant impact on nervous system disorders and oncogenesis.

CRL7 and CRL9 are more recently identified CRLs. Only two F-box proteins, FBXW8 and FBXW11, have been identified as the substrate receptors for CRL7. The CRL7 complex, composed of the Cul7 scaffold, the RBX1 RING protein, and the SKP1 adaptor, is known to mediate both proteolytic and non-proteolytic ubiquitination. Its substrates are involved in the regulation of cell proliferation, apoptosis, and DNA damage repair [8]. The exact components of CRL9 are largely unknown, but it has been reported to mediate the ubiquitination and degradation of survivin and to maintain genome integrity [9]. Consequently, CRL9 is considered a tumor suppressor due to its function in regulating survivin and p53.

HECT domain E3 ligase is the second largest family, which possesses a conserved HECT domain containing a catalytic cysteine residue [10]. This domain functions as the catalytic core, mediating the direct transfer of ubiquitin to the substrate. HECT E3 ligases facilitate ubiquitination through a distinctive two-step catalytic mechanism. First, ubiquitin is transferred from the E2 ubiquitin thioester intermediate to a conserved catalytic cysteine within the HECT domain, forming a transient E3 ubiquitin thioester intermediate. In the second step, the ubiquitin moiety is conjugated to a specific lysine residue on the substrate protein via an isopeptide bond, resulting in stable substrate ubiquitination.

NEDD4 (neural precursor cell expressed developmentally down-regulated 4) family is a well-characterized HECT E3 ubiquitin ligase, which plays a critical role in diverse cellular processes including membrane trafficking, signal transduction, and protein degradation. Importantly, several HECT E3 ligases exhibit context-dependent functions in cancer, acting as either oncogenes or tumor suppressors depending on the specific substrates they target and the cellular environment. For instance, NEDD4 has been shown to function as a tumor promoter in colorectal cancer, highlighting the complexity of its role in tumorigenesis [11,12]; whereas, NEDD4L functions as a tumor suppressor by controlling Wnt signaling pathway. Similarly, other members of the HECT E3 family, such as HECTD3 and HUWE1, have also demonstrated the ability to play dual roles across different cancer types, reinforcing the importance of cellular context in determining their function [13,14].

U-box E3 ubiquitin ligases are the smaller group among other ubiquitin ligases. U-box E3 ubiquitin ligases possess a conserved U-box domain in their C-terminal domain from yeast to humans and play critical roles in several cellular processes [15]. Interestingly, they utilize the intramolecular interactions for its scaffold function. Further, they are prevalent in plants compared with the animals [16].

RBR domain E3 ligases exhibit features of both RING and HECT ligases. RBR ligases typically include three consecutive structural domains: an N-terminal classical RING (RING1) domain, a central in-between RING (IBR) domain, and a C-terminal RING2 domain, which is also referred to as the Rcat (‘required-for-catalysis’) domain [17]. Some RBRs may also possess a central BRcat (‘benign-catalytic’) domain, which adopts a similar fold to Rcat but lacks catalytic activity. The RING1 domain engages with a ubiquitin-loaded E2 enzyme, a mechanism similar to the RING E3 ligases. Subsequently, ubiquitin is transferred from the E2 to a catalytic cysteine residue located within the RING2 (Rcat) domain of the RBR ligase, forming a thioester bond. Finally, the RBR ligase directly conjugates this ubiquitin to the substrate protein. Parkin (PARK2) is a well-characterized RBR E3 ligase, mostly functions as a tumor suppressor. It has been shown to inhibit primary and metastatic tumor growth, shutting off mitochondrial dynamics and inhibiting the non-oxidative phase of the pentose phosphate pathway, thereby blocking tumor cell movements [18]. The structural and mechanistic diversity of E3 ligase families suggests a wide range of regulatory functions and potentially different approaches for therapeutic targeting. E3 ligases decide which substrate is needed to be ubiquitinated. Therefore, deregulation of E3 ubiquitin ligases results in alteration of turnover kinetics of proteins, leading to cellular processes impairment associated with diseases. Thus, they are attractive targets for therapeutic intervention, as they offer the potential to modulate specific pathways without broadly affecting the UPS.

## E3 ubiquitin ligases in the hallmarks of cancer metastasis

Cancer metastasis is a highly complex and multi-step biological process during which cancer cells detach from the primary tumor and establish new, secondary tumors in the distant organs. Epithelial cancer cells acquire migratory and invasive properties through EMT, the first step of metastasis.

Once transformed, cancer cells degrade the surrounding extracellular matrix to enable movement and invasion into adjacent tissues. Following local invasion, these cells penetrate the walls of nearby blood vessels (capillaries or venules) or lymphatic vessels, thereby entering the circulatory or lymphatic system. Within these systems, circulating tumor cells (CTCs) encounter numerous physiological challenges that limit their survival.

Only a small subset of CTCs successfully completes the next critical step, i.e. adhering to the endothelium at distant sites. These cells then extravasate by breaching the vessel wall and infiltrate the parenchyma of the target organ. To establish a secondary tumor, the extravasated cancer cells must adapt to the new microenvironment, evade local immune defenses, and proliferate. This final stage, known as colonization, is often the rate-limiting step of metastasis. Previous studies showed that E3 ubiquitin ligases play significant roles in each of these stages.

### Epithelial-to-mesenchymal transition (EMT)

EMT of cancer cells is the earliest process for the onset of cancer metastasis. Epithelial cells lose their cell–cell adhesion and polarity ability to gain mesenchymal characteristics during EMT, enabling them to migrate and invade. During this process, several proteins are ubiquitinated. Thus, E3 ligases have been reported to either promote or inhibit this EMT process. For instance, S-phase kinase-associated protein-2 (Skp2), a member of F-box family protein and highly up-regulated in many cancers, a crucial component of the SCF (Skp1-Cullin-F box) substrate recognition complex, is reported to promote the EMT through controlling the expression of several EMT marker proteins in multiple cancers [19–21]. Similarly, NEDD4 promotes EMT in colon cancer via the ubiquitination and degradation of FOXA1, a known transcription factor that inhibits EMT. Additionally, NEDD4 degrades the tumor suppressor PTEN to promote cancer stem cell (CSC) invasion and metastasis [11]. Also, TRIM31 promotes EMT via anoikis resistance by mediating K48 linked ubiquitination of p53 and hence AMPK pathway overactivation [22].

Tumor-promoting E3 ligase SMURF1 is associated with malignant phenotypes and promotes metastasis through activating EMT process. Several studies showed that it facilitates proteasome-mediated degradation of RhoA, DAB2IP (disabled homolog 2 [DAB2] interacting protein), TRAF4 (tumor necrosis factor receptor-associated factor 4), Kindlin2, talin-H to promote EMT process [23–25]. SMURF1 is also known to promote gastric cancer cell metastasis by activating the PI3K/AKT pathway to promote EMT process [26].

Another E3 ubiquitin ligase UBE3C is reported to promote melanoma through enhancing EMT [27]. It was shown that UBE3C increases the expression of vimentin and Snail1 and decreases the expression of E-cadherin. Another study reported that NEDD4-binding protein 3 (N4BP3) augments breast cancer metastasis through promoting EMT [27]. Mechanistically, it was observed that N4BP3 promotes E-cadherin degradation via ubiquitin ligase NEDD4-mediated ubiquitination [28]. In addition, it is reported that UBR5 drives EMT in triple-negative breast cancer (TNBC) by promoting degradation of E-cadherin [29]. In contrast, tumor suppressor F-box protein FBXW7, frequently in several cancers, inhibits EMT by promoting the ubiquitination and degradation of ZEB2 and mTOR, which are known to induce EMT in colorectal cancer cells [30]. Conversely, mutations in FBXW7 in non-small lung cancer promote EMT through stabilization of several EMT-associated proteins including TWIST-1 and SNAIL-1 [31]. Thus, FBXW7 plays a crucial role in preventing EMT through multiple ways. Like FBXW7, E3 ubiquitin ligase RNF128 (GRAIL) also prevents EMT process through abrogating Wnt/β-catenin signaling pathway [32]. Mechanistically, it was shown that down-regulation of RNF128 activates Wnt/β-catenin signaling that facilitates EMT through controlling the expression of CD44 and cortactin.

Tripartite motif-containing family proteins play a critical role in the EMT process through controlling the expression of Snail. It is reported that tripartite motif containing 16 (TRIM16) and 21 (TRIM21) degrade Snail to prevent EMT and block metastasis progression of colorectal cancer [33]. Further, authors showed that TRIM21 mediated down-regulation of Snail is prevented by EDAR-associated death domain (EDARADD) to promote colon cancer metastasis. Mechanistically, it was shown that EDARADD down-regulates the expression of TRIM21 both at the transcriptional level as well as at the post-translational level. Further, it is reported that TRIM62 also controls the expression level of Snail to prevent EMT. Herein, authors have shown that TRIM62 represses the expression of Snail through repressing the TGF-β-SMAD3 signaling pathway [34]. While a previous study showed that TRIM11 promotes the EMT process in hepatocellular carcinoma [35].

In addition to TRIM family members, a previous study showed that E3 ubiquitin ligase NEDD4L (neural precursor cell expressed developmentally down-regulated 4-like) inhibits EMT process to prevent gastric cancer progression [36]. NEDD4L inhibits the EMT process through controlling the expression of Bicaudal C homolog 1 (BICC1) at the proteasomal level in GC cells. Further, they found a positive correlation between BICC1 and AKT1. Thus, NEDD4L might prevent EMT through controlling the BICC1-AKT axis [36]. Low-level expression of NEDD4L in gastric cancer is associated with aggressive metastatic disease and poor clinical outcomes, suggesting its role in the inhibition of metastasis through preventing EMT. Mechanistically, it was found that HIF-1α might be responsible for low-level expression of NEDD4L in gastric cancer [37]. The contrasting roles of NEDD4 and NEDD4L in gastric cancer metastasis highlight the complexity within the E3 ligase families and the requirement for specific targeting strategies.

### Invasion and migration

Cancer cell invasion and migration are positively correlated with cancer metastasis. Therefore, the highly invasive and migratory nature of cancer cells counts for more metastatic potential. Several studies reported that E3 ligases also play crucial roles in regulating the invasive and migratory capabilities of cancer cells. For instance, Skp2 is reported to promote the invasion via enhancing the expression of MMP-2 and MMP-9 in lung cancer oral squamous cell carcinoma [38]. Similarly, Skp2 also increases the expression of MMP-2 and MMP-9 to promote invasion of lung cancer cells [39]. The high-Skp2 expression group had a poor prognosis as compared with the low-expression group.

Another study reported that ubiquitin ligase c-CBL prevents the migration and invasion of glioma cells by degrading αPix (also known as at Cool1) at the post-translational level [40]. A previous study reported that αPix, a member of guanine nucleotide exchange factors (GEFs), activates several Rho GTPase family proteins and actin cytoskeleton to promote cell invasion and migration.

Ubiquitin ligase TRIM31 promotes K48-linked and K63-linked ubiquitination of TSC2, leading to the activation of mTORC1, hence promoting the invasion and migration in hepatocellular carcinoma. Conversely, it promotes K63-linked ubiquitination of p53, inhibiting invasion and migration in breast cancer [22]. In addition to TRIM31, TRIM47 promotes cell proliferation, invasion, and migration of gastric cancer cells [22]. Authors found that TRIM47 promotes K48-linked ubiquitination and degradation of CYLD to activate NF-κB pathway and thereby promotes cell migration and invasion. Thus, higher expression of TRIM47 is linked with poor survival in non-small cell lung cancer and gastric cancer [41]. Another study reported that TRIM65 promotes tumor proliferation and invasion via the ubiquitin-mediated degradation of Rho GTPase-activating protein 35 (ARHGAP35) [42]. ARHGAP35 is a member of the Rho GAP family and it inhibits Rho GTPase to prevent cytoskeleton remodeling during cell migration and invasion [43].

A previous study showed that ubiquitin ligase MDM2/MDMX promotes cell migration and invasion through controlling the expression of Sprouty4 [44]. Mechanistically, it was shown that MDM2 directs the degradation of Sprouty4 to increase the expression of RhoA for augmenting cell migration and invasion.

Ubiquitin ligase Parkin is reported to act as a tumor suppressor in bladder cancer (BLCA) by inhibiting cell migration [45]. Authors found that Parkin degrades Catalase to attenuate the ROS generation-mediated cell migration. In contrast, highly expressed USP30, a substrate of Parkin, deubiquitinates Catalase to promote cell migration in BLCA cells [45]. Another study demonstrated that FBXO28 inhibits invasion and migration in hepatocellular carcinoma via PKA-dependent ubiquitination and degradation of Slug [46]. SMURF2 inhibits the invasion and migration of breast cancer cells by degrading SMURF1 [47]. They found that SMURF2-mediated degradation of SMURF1 results in activation of TGFβ signaling, leading to repression of cell invasion and migration [47].

Several genes like *VANGL1, VANGL2, and FZD7* are involved in planar cell polarity, and they play a critical role in Wnt signaling to promote cell invasion and migration of cancer cells. It was observed that *VANGL1* and *VANGL2* are highly up-regulated in glioblastoma (GBM) progression and promote cell invasion and migration [48]. Wald et al. found that NRDP1 inhibits the Wnt signaling pathway to prevent GBM cell invasion and migration. They have shown that NRDP1 interacts with Vangl1 and Vangl2 to promote the K63-linked ubiquitination of Dishevelled to prevent its localization to the membrane and thereby it prevents Wnt signaling.

Another E3 ligase, SPOP (Speckle-type POZ protein), inhibits migration and invasion of prostate cancer cells by directing proteasomal degradation of Nanog. Authors found that SPOP mutation in prostate cancer is associated with high level nanog expression and cell invasion [42]. Hakai is an E3 ubiquitin ligase known for the proteasome-mediated degradation of E-cadherin. Its overexpression decreased the expression of Paxillin component, which is essential for focal adhesions. A decrease in the expression of Paxillin and degradation of E-cadherin results in reduced cell-substratum adhesion and increased epithelial cell invasion [49].

Ubiquitin ligase HUWE1 promotes migration and invasion in gastric cancer by directing proteasomal degradation of ubiquitination and degradation of tumor suppressor TGFBR2 [50]. This highlights the context-dependent role of TGF-beta signaling in cancer progression, as its down-regulation via HUWE-1 promotes metastasis. In HGF-treated lung cancer cells, HUWE1 ubiquitinates the TIAM1 cell-cell junction, which leads to the disassembly of the cellular junction and an increase in cell invasion and metastasis [51].

Ubiquitin ligase HACE1 inhibits cell migration by mediating the down-regulation of β-catenin expression leading to abrogation of Wnt signaling. Its level getting significantly reduced is associated with poor overall survival in early-stage gastric cancer patients [52]. Further, HACE1 also inhibits the migration of colorectal cancer [53]. Authors reported that HACE1 markedly attenuates the expression of YAP1 in different colorectal cells.

### Angiogenesis

The formation of new blood vessels, or angiogenesis, is essential for tumor growth and metastasis as it provides the necessary nutrients and oxygen for cancer cells at both primary and secondary sites. Parkin targets HIF-1α for ubiquitination and degradation, preventing breast cancer cell migration and invasion [54]. c-CBL, a cytosolic RING finger domain ubiquitin E3, inactivates PLCγ1 (phospholipase Cγ1) by ubiquitination in endothelial cells and inhibits VEGF-driven angiogenesis [55]. Although it is not specifically related to angiogenesis, HIF-1α is a key regulator of angiogenesis, suggesting a potential indirect role of Parkin in the inhibition of HIF-1α. These findings suggest that E3 ligases can influence angiogenesis, a critical process associated with metastasis, through direct or indirect regulation of key factors like HIF-1α and VEGF (implied by the role of cytokines in angiogenesis). Another study reported that TRIM24 and TRIM28 regulate angiogenesis through controlling VEGF expression. However, future studies are needed to understand the molecular mechanism of regulation of VEGF by TRIM24 and TRIM28 [56].

### Colonization

For metastasis to be successful, cancer cells reach distant sites, survive, and grow to form secondary tumors, a process known as colonization. RNF128 (GRAIL) induces CSC turnover by ubiquitinating and degrading CD44/CTTN in melanoma [32]. This role in regulating CSC turnover suggests its involvement in controlling the population of cells with metastatic potential. While Parkin targets HIF-1α, which can also influence survival in distant niches, its direct role in regulating CSC invasion and metastasis requires further study. The role of E3 ligases in the colonization stage of metastasis, particularly in regulating cancer stem cells, requires further investigation.

## Therapeutic potential of targeting E3 ubiquitin ligases in cancer metastasis

E3 ubiquitin ligases control protein stability. Therefore, deregulation of ubiquitin ligases is closely associated with cancer progression and metastasis. Hence, there have been several inhibitors developed to control the function of E3 ubiquitin ligases to regulate cancer metastasis. These inhibitors are classified into two major groups as discussed below.

### Direct inhibition of E3 ligase activity

This involves developing small-molecule inhibitors (SMIs) to block the function of specific oncogenic E3 ligases.

### MDM2 inhibitors

Ubiquitin ligase MDM2 is known to inactivate tumor suppressor p53 by facilitating its proteasomal degradation. Several small molecules are developed to activate p53 through disruption of the MDM2-p53 axis, and some of them have successfully entered clinical trials (Phase I–III) [57–64]. However, challenges include dose-limiting hematological toxicities and acquired resistance, necessitating combination therapy approaches.

### SCF inhibitors

Developing small molecule inhibitors for oncogenic F-box proteins like Skp2 or β-TrCP is very challenging as they also play a vital role in maintaining the cellular homeostasis of normal cells. Nevertheless, several attempts have been made to develop inhibitors against SKP2, and few inhibitors have entered and successfully progressed through pre-clinical trials. For instance, it is reported that Skp2 inhibitor C1 (SKPin C1) potently suppresses the growth of several cancers [65–68]. Mechanistically, it was observed that SKPin C1 prevents SKP2-mediated degradation of p27 through perturbation of SKP2-p27 interaction. Another inhibitor of SKP2, SZL-P1-41, is also found to inhibit the cancer progression and reduce the cancer stem cell population [69]. It shows potent antitumor activities in multiple animal models, further establishing it as a therapeutic strategy in preclinical settings. It was also observed that SZL-P1-41 potently inhibits the interaction of SKP2 and SKP1 to prevent the SKP2 ubiquitin ligase activity.

Of note, neddylation of cullin proteins is obligatory for function of CRL complexes. Ubiquitin-like protein NEDD8 is covalently attached to the cullins during neddylation [70,71]. Therefore, MLN4924 is developed to inhibit the neddylation to inactivate CRL complexes for modulating cancer progression. Wherein, it was shown that MLN4924 inhibits the NEDD8-activating enzyme (NAE), thereby preventing the neddylation of cullin proteins [72]. This upstream inhibition strategy is another important avenue of research for targeting CRL complexes.

### Targeted protein degradation via PROTACs

Proteolysis-targeting chimeras (PROTACs) are heterobifunctional molecules that hijack specific E3 ligases (commonly VHL or Cereblon/CRBN) to induce the ubiquitination and subsequent proteasomal degradation of a target protein of interest (POI), often an oncoprotein. This catalytic event-driven mechanism offers potential advantages over traditional inhibitors, including the ability to target ‘undruggable’ proteins with potentially lower toxicity. Several PROTACs are developed to target cancer drivers like the androgen receptor (AR) and estrogen receptor (ER), and they have entered phase I/II clinical trials for metastatic prostate and breast cancer, respectively, showing encouraging signs of target degradation and clinical activity. Challenges include optimizing drug-like properties (delivery, bioavailability), ensuring selectivity, and overcoming potential resistance mechanisms (e.g., mutations in the POI or the recruited E3 ligase) [73,74].

Overall, targeting E3 ubiquitin ligases holds significant therapeutic potential to combat cancer metastasis. However, to translate it into the clinic requires addressing the challenges such as specificity, toxicity, resistance, and delivery. Therefore, future studies are needed to overcome these issues.

## Conclusions

**Figure 1:**
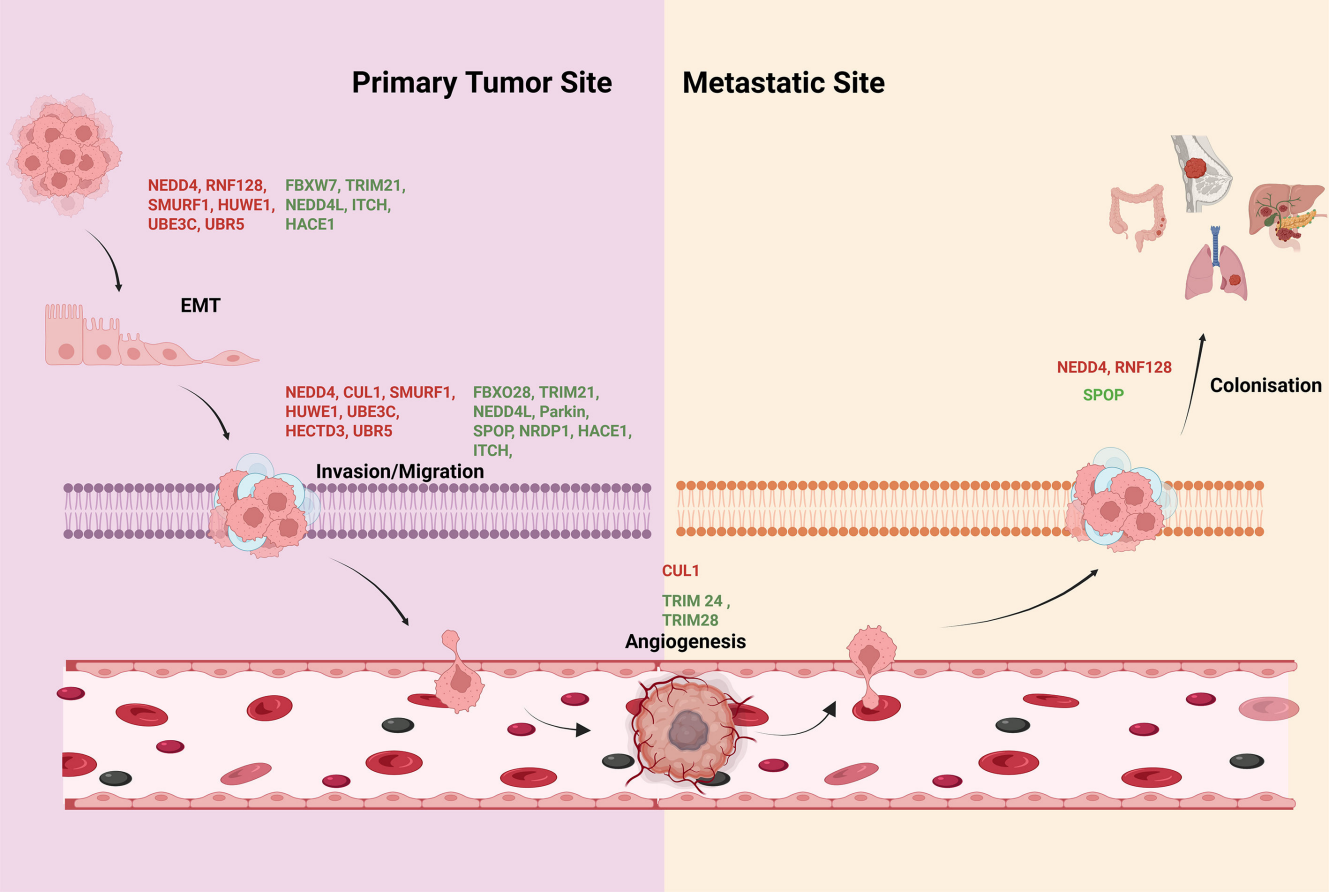
Schematic representation showing the role of E3 ubiquitin ligase in the regulation of hallmarks of cancer metastasis. Green color depicts the E3 ubiquitin ligase which inhibits the metastasis and red color indicates the promotion of cancer metastasis.

E3 ubiquitin ligases are critical players in maintaining cellular homeostasis and integrity. Therefore, deregulation of E3 ubiquitin activities is associated with several diseases including cancer. They are involved in cancer initiation to metastasis. Here, we reviewed the importance of E3 ubiquitin ligases in different steps of metastasis as shown in Figure 1. Several ubiquitin ligases inhibit metastasis, while others promote metastasis through controlling different steps of metastasis (Table 1). Therefore, metastasis-promoting E3 ubiquitin ligases might be potential targets to develop cancer therapeutics.

**Table 1:** Specific E3 ubiquitin ligases and their roles in different stages of cancer metastasis

E3 ligase name	Cancer type(s)	Role in metastasis	Specific stage(s) affected	Key downstream targets
NEDD4	Gastric, Breast (CSCs)	Promotes	EMT, Invasion, Migration, Colonization	FOXA1, PTEN
FBXW7	Colorectal	Inhibits	EMT	mTOR, TWIST-1, SNAIL-1
FBXO28	Liver	Inhibits	Invasion and migration	SNAI2
RNF128 (GRAIL)	Melanoma	Promotes	EMT, Colonization	CD44/CTTN
CUL1	Breast	Promotes	Migration, Invasion, Angiogenesis	EZH2, CXCL8, IL11
SMURF1	Gastric	Promotes	EMT, Invasion, Migration	DAB2IP
HUWE1	Gastric	Promotes	EMT, Migration, Invasion	TGFBR2
UBE3C	Gastric	Promoting	EMT, Invasion, Migration	AXIN1
TRIM21	Breast	Inhibits	EMT, Invasion, Migration	Snail
NEDD4L	Gastric	Inhibits	EMT, Invasion, Migration	BICC1, PI3K/AKT , HIF-1α
ITCH	Gastric	Inhibits	EMT, Invasion, Migration	β-catenin, p-Dvl2
HACE1	Gastric	Inhibits	EMT, Migration, Invasion	β-catenin
c-CBL	Glioma, Lung, Gastric	Context-dependent	Invasion, Migration	αPix, EGFR
NRDP1	Glioblastoma	Inhibits	Invasion, Migration	DVL2
SPOP	Prostate	Inhibits	Invasion, Migration, Colonization	Nanog, BRD4
HECTD3	Breast, Gastric	Promotes	Invasion, Migration	MALT1
UBR5	Gastric, Breast	Promotes	EMT, Invasion, Migration	GKN1, E-cadherin
Parkin	Breast	Inhibits	Migration, Invasion, Angiogenesis (indirect)	HIF-1α

Intravasation and extravasation steps are very critical steps in cancer metastasis. Though cell migration and invasion play a critical role in the intravasation and extravasation, how cancer cells invade the epithelial barrier of blood vessels is not well understood. Therefore, future studies are needed to identify the E3 ubiquitin ligases that control intravasation and extravasation steps. Thus, a detailed understanding of these research gaps in metastasis might pave the way for the development of more effective and targeted therapies to combat this devastating aspect of cancer progression.

**Summary Points**

- E3 ubiquitin ligases are important for tagging proteins specifically for degradation and functional modification. Their dysregulation, i.e., mutation and altered expression, is often observed in cancer.
- E3 ligases affect almost every stage of metastasis, including the EMT, invasion and migration, angiogenesis, interactions with the tumor microenvironment (including immune evasion and response to hypoxia), and successful colonization at distant sites.
- Recent strategies involve the development of small-molecule inhibitors to block E3 activity (e.g., MDM2 inhibitors like Nutlins, Navtemadlin, and Milademetan, which show activity in specific cancers but face toxicity/resistance issues) and utilizing Proteolysis-Targeting Chimeras (PROTACs) to hijack E3 ligases (like VHL or CRBN) to promote the degradation of oncogenic proteins (e.g., AR/ER degraders showing promise in clinical trials).
- The E3 ligases have context-dependent functions and show significant cross-talk among ligases for cancer metastasis. Challenges like inhibitor specificity, drug resistance, delivery, and identifying predictive biomarkers, along with exploring rational combination therapies, are key future directions for successfully translating E3-targeted therapies into effective anti-metastatic treatments.

## Future perspective

Human genome encodes approximately 600 E3 ubiquitin ligases. Though a larger number of the ubiquitin ligases are dysregulated in cancer, only a few E3 ubiquitin ligases are explored for their role in cancer. Therefore, detailed future studies are needed to understand the role of dysregulated E3 ubiquitin ligases in cancer progression. Further, targeting E3 ubiquitin ligases holds significant therapeutic potential to combat cancer metastasis. However, to translate it into the clinic requires addressing the challenges such as specificity, toxicity, resistance, and delivery. Therefore, future studies are needed to overcome these issues.

## Open Access

This article has been published open access under our Subscribe to Open programme, made possible through the support of our subscribing institutions, learn more here: https://portlandpress.com/pages/open_access_options_and_prices#conditional

## Abbreviations

CTCs: circulating tumor cells
ECM: extracellular matrix
EMT: epithelial-to-mesenchymal transition
UPS: ubiquitin–proteasome system
